# Smoking by family members and friends and electronic-cigarette use in adolescence: A systematic review and meta-analysis

**DOI:** 10.18332/tid/84864

**Published:** 2018-02-27

**Authors:** Jian-Wei Wang, Shuang-Shuang Cao, Ru-Ying Hu

**Affiliations:** 1 Yidu Central Hospital of Weifang, Qingzhou, Shandong, China; 2 Zhejiang Provincial Center for Disease Control and Prevention, Hangzhou, Zhejiang, China

**Keywords:** electronic cigarettes, cigarette smoking, adolescents, metaanalysis

## Abstract

**INTRODUCTION:**

Evidence suggests that smoking by family members and friends is a strong predictor of smoking uptake in adolescents, yet the influence on electronic cigarette (e-cigarette) use has not been systematically reviewed and quantified.

**METHODS:**

Relevant studies were identified by searches of the PubMed and ScienceDirect databases up to December 2016. The summary odds ratios (ORs) with 95% confidence intervals (CIs) were calculated using a random-effects model.

**RESULTS:**

A total of 21 studies were included in this meta-analysis. A positive association was observed between adolescent e-cigarette use and smoking by family members (OR=1.47, 95% CI=1.30-1.66) and friends (OR=2.72, 95% CI=1.87-3.95), even after adjusting for the individual smoking status. Stratified by family members, the association with smoking in siblings (OR=1.87, 95% CI=1.35-2.60) was more prominent than that in parents (OR=1.41, 95% CI=1.191-.68) and other family members (OR=1.39, 95% CI=1.12-1.72).

**CONCLUSIONS:**

The present meta-analysis suggests that smoking by family members and friends is significantly associated with increased probability of e-cigarette use in adolescents.

## INTRODUCTION

Electronic cigarettes (e-cigarettes), the most common prototype of electronic nicotine delivery systems, are devices that do not burn or use tobacco leaves but instead vaporize a solution that the user then inhales^1^. E-cigarettes were invented in 2003, and are gaining popularity around the world. However, during the last decade, e-cigarettes have been embroiled in a controversy over the safety and efficacy for smoking cessation, as reliable evidence on these issues is limited and inconsistent. In recent years, it is worth noting that e-cigarette use has been increasing rapidly in adolescents in many countries^2^, and particular concerns have been raised on the potential health consequences of their use. E-cigarettes can influence the physical development of adolescents. It has been suggested that e-cigarette use may be associated with the increased prevalence of respiratory symptoms and asthma in children and adolescents, even after adjusting for the smoking status^3,4^. However, Polosa et al. found that e-cigarettes could be a protective factor in adult asthmatic people^5^. E-cigarettes may also serve as a gateway to smoking, with cross-sectional and longitudinal studies indicating that adolescents who used e-cigarettes were at risk for subsequent progression to conventional cigarette smoking^6,7^. However, some studies drew different conclusions and proposed that these findings should be interpreted cautiously due to methodological weaknesses^8,9^. In light of the high use of these devices and potential for health consequences, it is important to understand factors related to e-cigarette use in adolescents.

Since a history of cigarette smoking has identified the common correlate of e-cigarette use^10^, one way to explore the factors of adolescent e-cigarette use behaviors is to examine the established predictors of cigarette smoking in this age group. From this perspective, some studies have found that individual characteristics, including age, gender, socioeconomic status, school performance and family structure were significantly associated with the e-cigarette use in adolescents^11-13^. Besides, as smoking by family members and friends has been suggested as a strong predictor of smoking uptake in adolescents^14,15^, these family and peer influences may extend to the adolescent e-cigarette use behaviors. However, to date, these influences have not been systematically reviewed and quantified. Therefore, in this paper we retrieved the existing literature, extracted the relevant data and provided summary estimates of effects of smoking by family members and friends on e-cigarette use in adolescents.

## METHODS

### Literature and search strategy

This study followed the Preferred Reporting Items for Systematic Reviews and Meta-Analyses (PRISMA) statement^16^. We performed literature searches within two databases (PubMed, ScienceDirect) from 2003 to December 2016 to identify potentially relevant studies on the association between smoking by family members (parents, siblings, other family members) and friends and e-cigarette use in adolescents (10-19 years old). The literature search was limited to the English language. The outcome of e-cigarette use was defined empirically from those used in these studies and the current e-cigarette use was used in preference where available. Detailed definitions of e-cigarette use are shown in the Supplementary Table S1. To identify terms related to family member and friend smoking, we combined the following keywords with OR: ‘mother’, ‘father’, ‘parent’, ‘parental’, ‘sibling’, ‘family’, ‘household’, ‘family member’, ‘friend’, ‘peer’, ‘smoking’, ‘smoking exposure’, ‘cigarette smoking’, and ‘tobacco use’. Relevant outcomes were identified by searching keywords for ‘electronic cigarette’, ‘e-cigarette’, ‘electronic nicotine delivery systems’ and combining these with OR. Both exposure and outcome searches were combined with AND. Reference lists of retrieved literature were also screened to identify relevant articles.

### Inclusion and exclusion criteria

Studies had to investigate the association between smoking by family members and friends and e-cigarette use in adolescents and provide the adjusted odds ratios (AORs) with 95% confidence intervals (CIs) for family member and friend smoking or relevant data to calculate these. Studies from the same datasets by the same or different authors were not included in the meta-analysis.

### Data extraction

The dichotomous ORs with CIs were extracted directly or calculated using adjusted effect estimates from each study. Meanwhile, the following information was extracted from each study: 1) the first author’s name, 2) publication year, 3) data source and age distribution, 4) location of the study, 5) number of e-cigarette users and study population, 6) study type, 7) e-cigarette use definition, and 8) variables adjusted. Two authors independently assessed the articles and extracted the above information, with differences resolved by discussion. The quality of each eligible study was assessed using the 9-star Newcastle-Ottawa Scale^17^.

### Statistical analysis

Based on the heterogeneity between studies, a fixed-or random-effects model was used to calculate the pooled ORs with 95% CIs for family member and friend smoking, respectively. If there was significant heterogeneity, a random-effects model would be used to assign the weight of each study. If there was evidence of no heterogeneity, we used a fixed-effects model with effect estimates given equal weight to the inverse variance of the study. Possible heterogeneity between studies was assessed using Q-test and the I^2^ statistic^18^. For the Q-test, p<0.05 indicates significant level of heterogeneity. The I^2^ statistic represented the amount of total variation attributed to heterogeneity rather than chance. The low, moderate, and high degrees of I^2^ values were considered to be 25, 50 and 75 %, respectively. To explore the possible source of heterogeneity between studies, subgroup analyses were conducted mainly based on different family members (parents, siblings, other family members), whether or not the individual smoking status was adjusted, and the geographic location of each study (Europe, Australia, Asia, America). Sensitivity analysis was conducted to test the stability of the present meta-analysis results. Publication bias was assessed by Egger’s regression asymmetry test^19^ (p< 0.05 was considered statistically significant). The Egger test detects funnel plot asymmetry by determining whether the intercept deviates significantly from zero in a regression of standardized effect estimates against their precision. All the statistical analyses were conducted with STATA Version 11 software (StataCorp LP, College Station, TX, USA).

## RESULTS

### Search results

**Figure 1:**
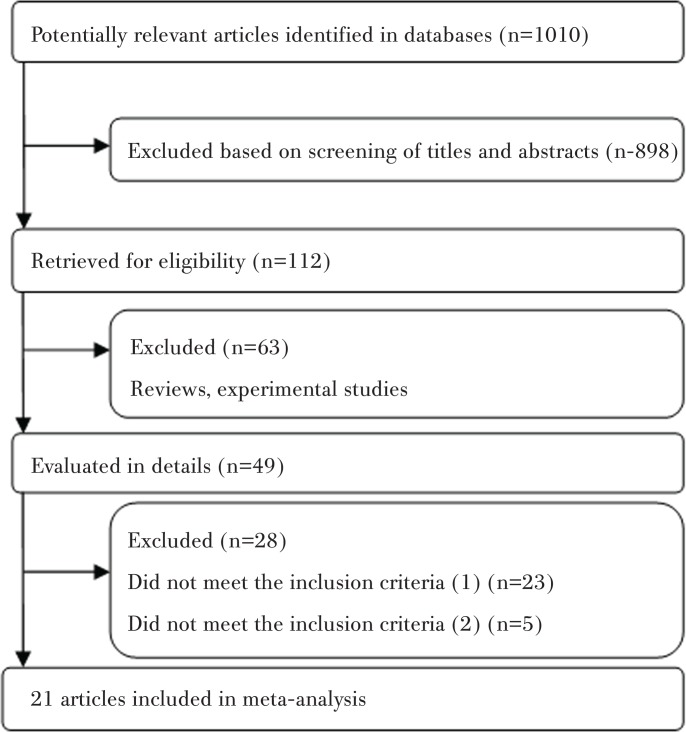
Flow chart of the study selection

Our initial search of the databases identified 1010 relevant articles, and 898 were excluded after screening the titles and abstracts. Among the remaining 112 articles retrieved for eligibility, 63 articles were excluded because they were review and experimental studies. From the remaining 49 articles, a further 28 articles that did not meet the inclusion criteria were also excluded. Finally, a total of 21 articles were included in this meta-analysis. The flowchart of study selection is shown in Figure 1.

### Study characteristics

The 21 eligible studies included 241 926 participants, of which 12 805 were ever or current e-cigarette users. There were a total of 10 studies from Europe, 6 from America, 4 from Asia and 1 from Australia, with all included studies reporting adjusted effect estimates. The quality score of studies ranged from 4 stars to 8 stars, according to the 9-star Newcastle-Ottawa Scale. The main characteristics of these selected studies are shown in Supplementary Table S1.

### Meta-analysis

#### Family member smoking and e-cigarette use in adolescents

**Figure 2:**
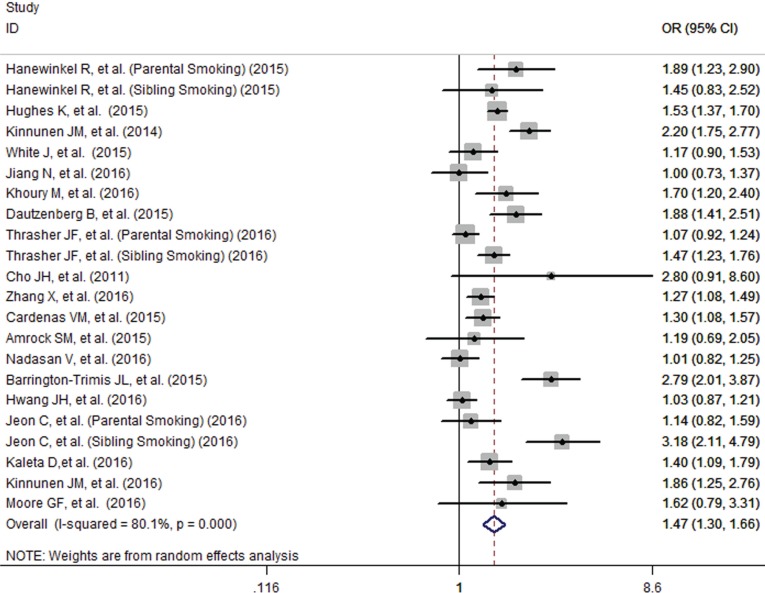
Influence of family member smoking on e-cigarette use in adolescents

**Figure 3:**
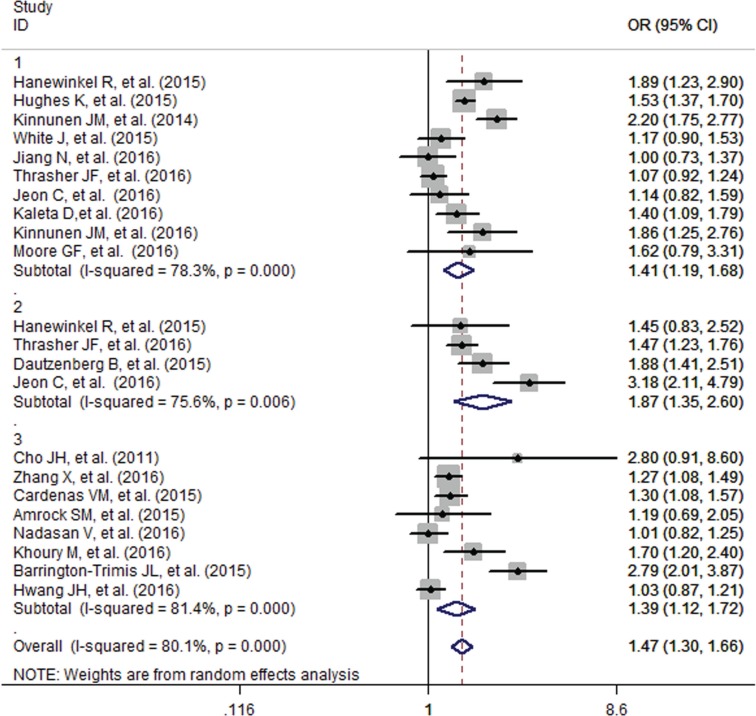
Specific influence of smoking on adolescent e-cigarette use among different family members ( 1-parents, 2-siblings, 3-other family members)

**Figure 4:**
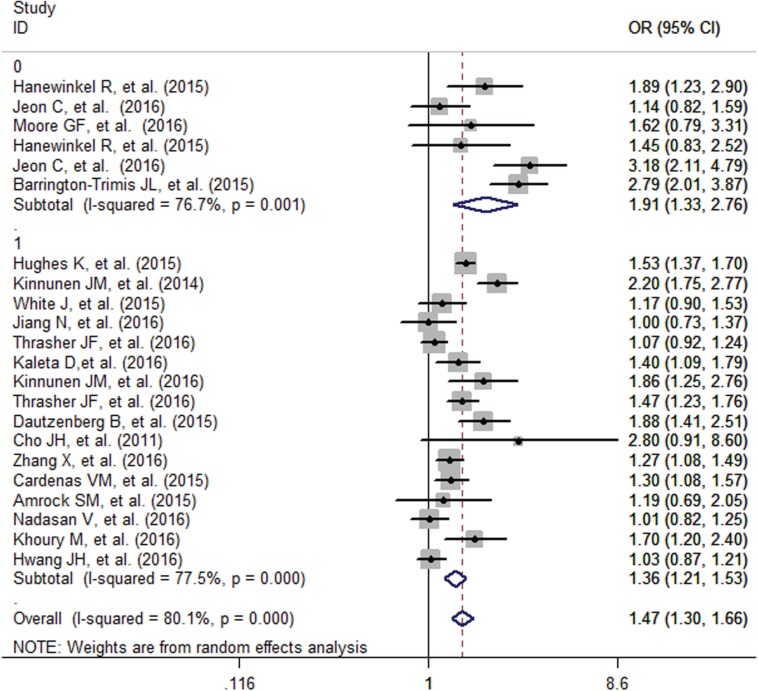
Influence of family member smoking on e-cigarette use in adolescents with or without adjusting for individual smoking status ( 0-No, 1-Yes)

The pooled analysis found that adolescents with smoking family members had an increased probability of e-cigarette use (OR=1.47, 95% CI=1.30-1.66), although the formal test for between-study heterogeneity gave a significant result (I^2^=80.1%, p<0.001) (Figure 2). Subgroup analysis based on different family members found that the effect was stronger for smoking by the siblings (OR=1.87, 95% CI=1.35-2.60) than that of the parents (OR=1.41, 95% CI=1.19-1.68) and other family members (OR=1.39, 95% CI=1.12-1.72) (Figure 3). Studies which adjust for the individual smoking status of adolescents found a decreased but significant pooled effect (OR=1.36, 95% CI=1.21-1.53) than for those who did not (OR=1.91, 95% CI=1.33-2.76) (Figure 4). Besides, stratified by geographic location, the pooled effects for Europe, Australia, Asia and America, were 1.59 (95% CI=1.34-1.90), 1.17 (95% CI=0.90-1.53), 1.45. (95% CI=0.96-2.19) and 1.44 (95% CI=1.19-1.75), respectively (Supplementary Figure S1).

#### Friend smoking and e-cigarette use in adolescents

**Figure 5:**
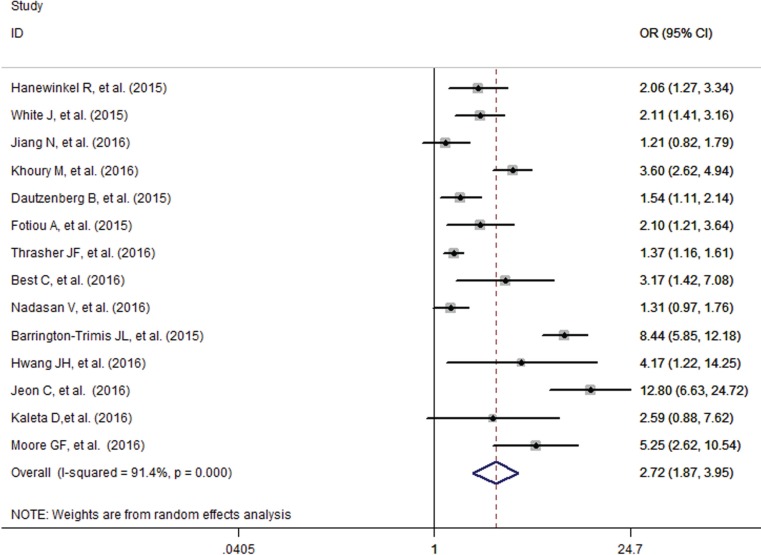
Influence of friend smoking on e-cigarette use in adolescents

**Figure 6:**
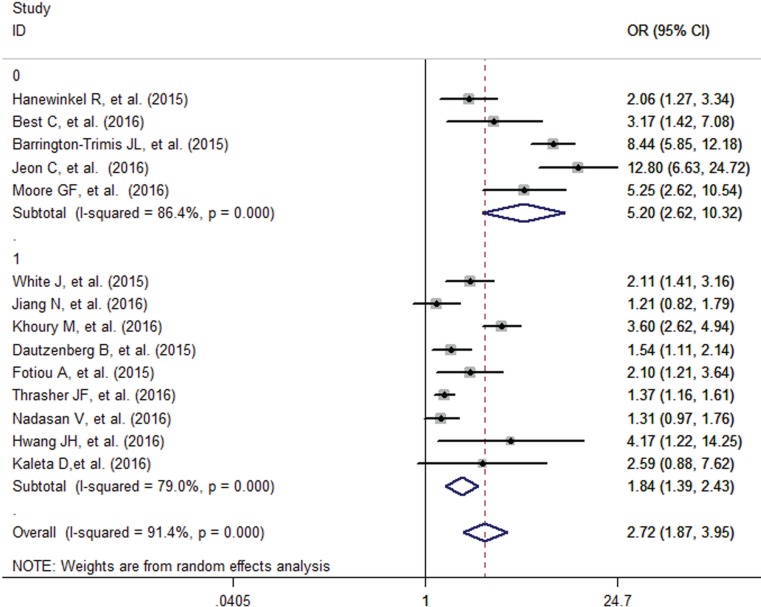
Influence of friend smoking on e-cigarette use in adolescents with or without adjusting for individual smoking status ( 0-No 1-Yes)

The pooled analysis found that adolescents having smoking friends were more likely to use e-cigarettes (OR=2.72, 95% CI=1.87-3.95), with high evidence of between-study heterogeneity (I^2^=91.4%, p<0.001) (Figure 5). Subgroup analysis found that studies that adjust for the individual smoking status of adolescents showed a decreased but significant pooled effect (OR=1.84, 95% CI=1.39-2.43) than that of those who did not (OR=5.20, 95% CI=2.62-10.32) (Figure 6). Stratified by geographic location, the pooled effects for Europe, Australia, Asia and America, were 2.11 (95% CI=1.51-2.94), 2.11 (95% CI=1.41-3.16), 3.95 (95% CI=0.72-21.67) and 3.43 (95% CI=1.16-10.13), respectively (Supplementary Figure S2).

#### Sensitivity analysis

To examine the stability of the observed significant associations, we conducted a sensitivity analysis by omitting each study from the analysis one at a time. For meta-analysis of family member smoking and e-cigarette use, the ORs were not dramatically changed and ranged from 1.42 (95% CI=1.27-1.60) to 1.50 (95% CI=1.32-1.70). The sensitivity analysis results are shown in Supplementary Figure S3. For meta-analysis of friend smoking and e-cigarette use, all the ORs were significant and ranged from 2.41 (95% CI=1.76-3.31) to 2.92 (95% CI=1.96-4.34). The sensitivity analysis results are shown in Supplementary Figure S4.

#### Publication bias

No evidence of publication bias was detected in the analysis of the association between e-cigarette use in adolescents and smoking by either family members (p=0.159) or friends (p=0.062).

## DISCUSSION

To our knowledge, the present study is the first comprehensive meta-analysis combining data from observational studies to investigate the possible effects of having family members and friends who smoke on an adolescent’s e-cigarette use behaviors. Overall, our results showed that family member and friend smoking is significantly associated with increased probability of e-cigarette use in adolescents, even after adjusting for the individual smoking status.

Our study has several strengths, including large sample size and relatively precise effect estimates adjusted for potential confounders in each study. However, some limitations of our study should be noted. First, the heterogeneity test shows high between-study heterogeneity in this meta-analysis. To explore the possible sources of heterogeneity, we conducted subgroup analyses mainly based on: different family members, whether or not the individual smoking status was adjusted, and geographic location of each study. However, a between-study heterogeneity that ranged from moderate to high was still observed in some subgroups, which suggests that other unknown confounding factors may be present. Secondly, most of the included studies are cross-sectional studies in the current meta-analysis, allowing no causal conclusions on the relationship between family member and friend smoking and e-cigarette use in adolescents. Thirdly, both exposure and outcome data in our study are based on adolescent self-reports, which may be susceptible to misreporting. Finally, smoking usually precedes e-cigarettes and our meta-analysis is not limited to non-smokers. Consequently, it may be difficult to prove smoking by family members and friends will influence e-cigarette use when the two behaviors are co-occurring.

There have long been international concerns, supported by growing research, that smoking by the key persons such as parents, as well as siblings and friends, can influence smoking uptake in adolescents^14,15^. Hence, our findings probably exacerbate public health concerns, by providing new evidence that a similar influence may extend to e-cigarette use behaviors. According to previous studies, the effects of family and friend smoking on adolescent smoking were thought to operate through genetics, behavior imitation, peer pressure and secondhand tobacco smoke exposure^20-23^. Similarly, we propose that these genetic and environmental factors may explain the observed family and peer influence on e-cigarette use in adolescents in the current study.

The family influence on smoking has been well established, and in general, the influence of parental smoking is considered the strongest predictor of adolescent smoking uptake^14^. In contrast, our observations of subgroup analysis indicate that smoking by siblings influences e-cigarette use more than that of parents and other family members. This has not yet been reported previously and the exact reasons are unknown. When family influence is compared to that of friends, the estimates from our study indicate that the influence of friends is stronger. Similar findings have been reported in a previous study, and a possible explanation is that peer influence on smoking behaviors appears to be more important during adolescence^15,24^. Besides, a recent Finnish study suggested that the most common source for e-cigarettes was friends, which may also partly explain the observed stronger influence of friends^13^. However, considering that adolescent smokers choose friends with similar smoking behaviors^24^, the peer influence in dual users (i.e. those who use cigarettes and e-cigarettes) should be interpreted with caution, and further longitudinal studies with nonsmoking adolescents are warranted to examine this proposition.

## CONCLUSIONS

In conclusion, the results of our meta-analysis suggest a significant positive association between family member and friend smoking and e-cigarette use in adolescents. Further evidence from prospective studies with nonsmoking adolescents is required to confirm these relationships.

## CONTRIBUTOR STATEMENT

Jian-Wei Wang and Shuang-Shuang Cao contributed to the work equally.

## CONFLICTS OF INTEREST

Authors have completed and submitted the ICMJE Form for Disclosure of Potential Conflicts of Interest and none was reported.
