# Supplementary figures and images for: Smoking by family members and friends and electronic-cigarette use in adolescence: A systematic review and meta-analysis
Source: Tob Induc Dis. 2018 Feb 27;16:05. doi: 10.18332/tid/84864 (PMC6659504; doi:10.18332/tid/84864)

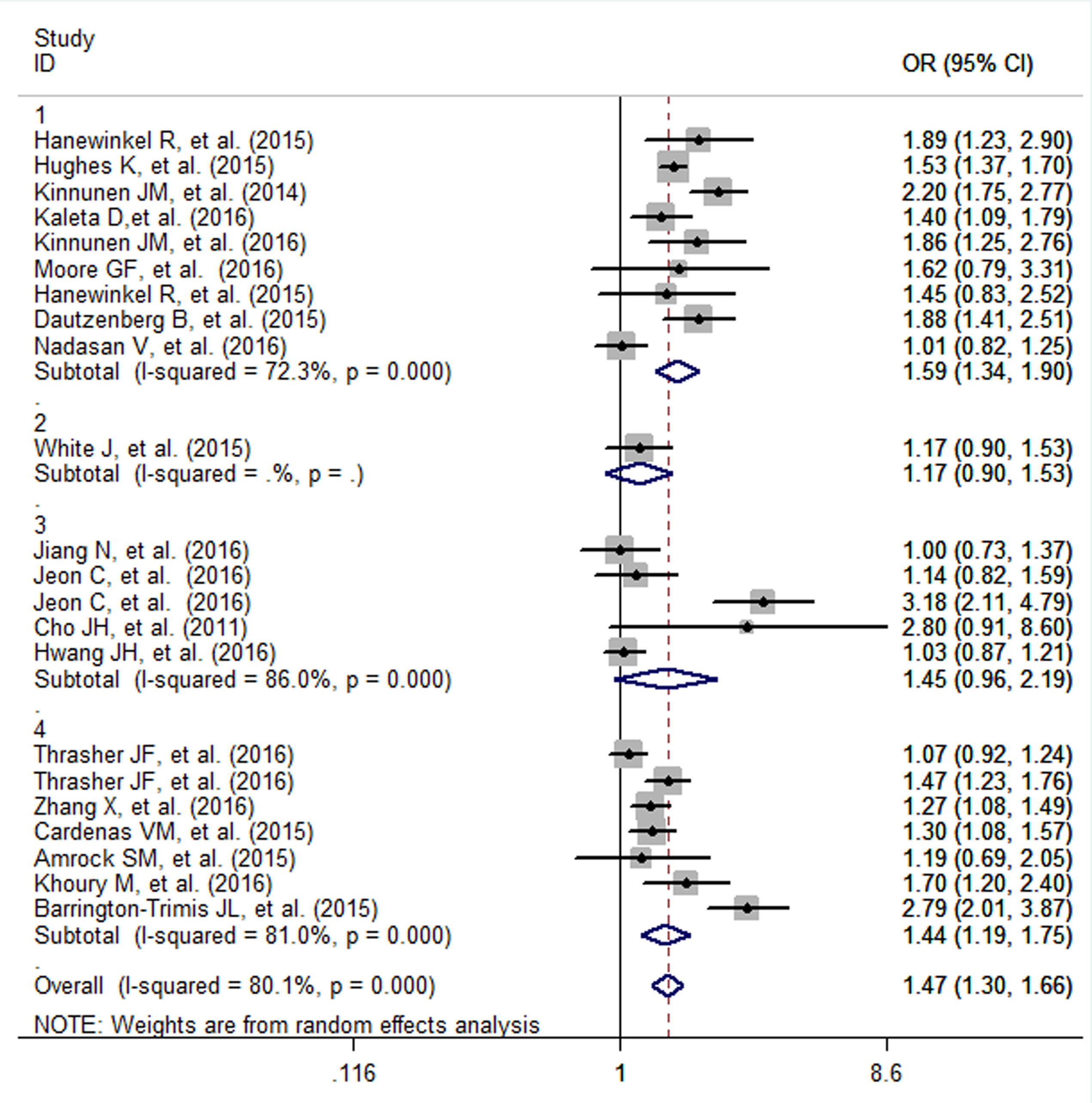

Supplement: Supplementary file 1 [file TID-16-05-s1.tif]

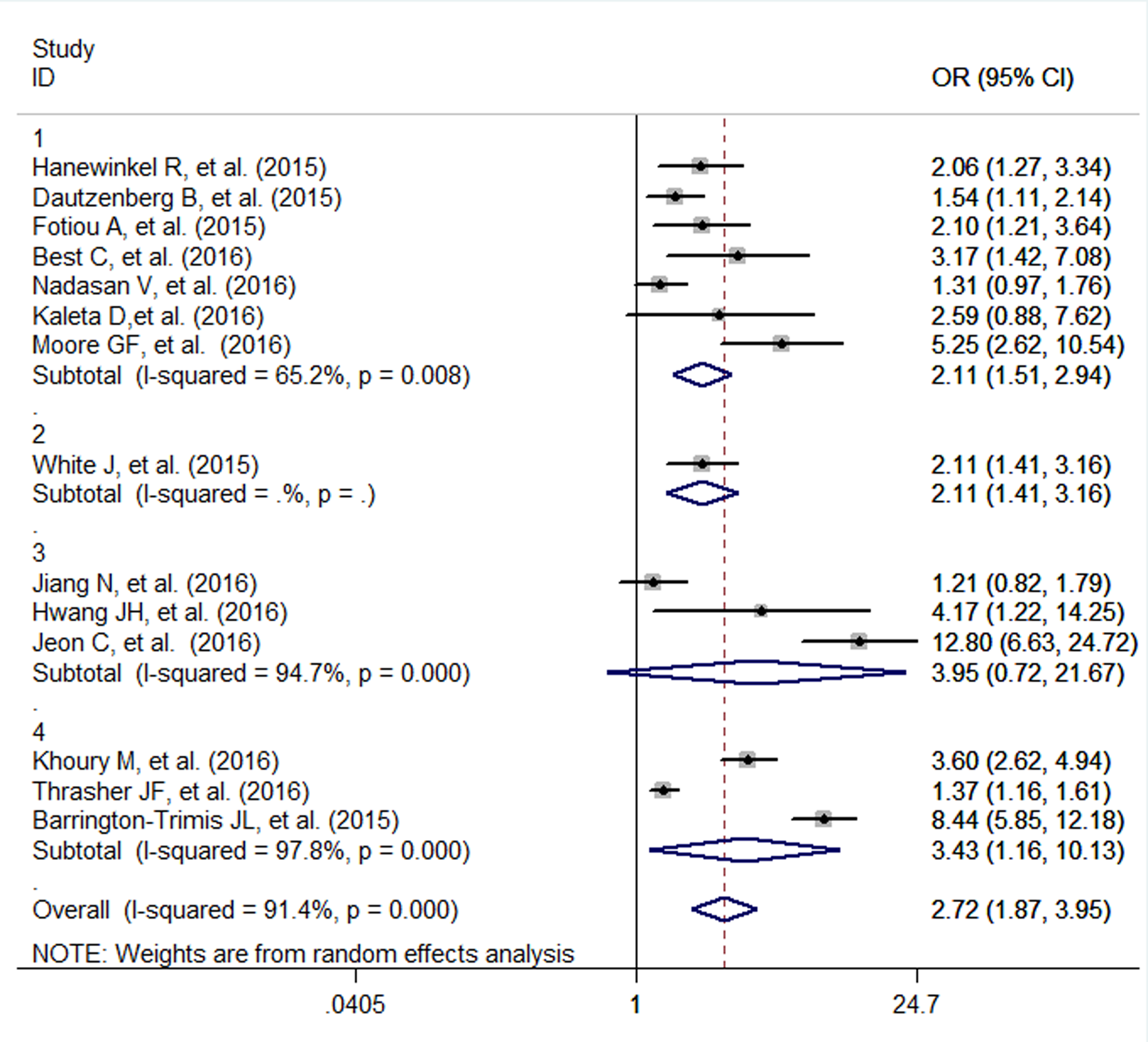

Supplement: Supplementary file 2 [file TID-16-05-s2.tif]

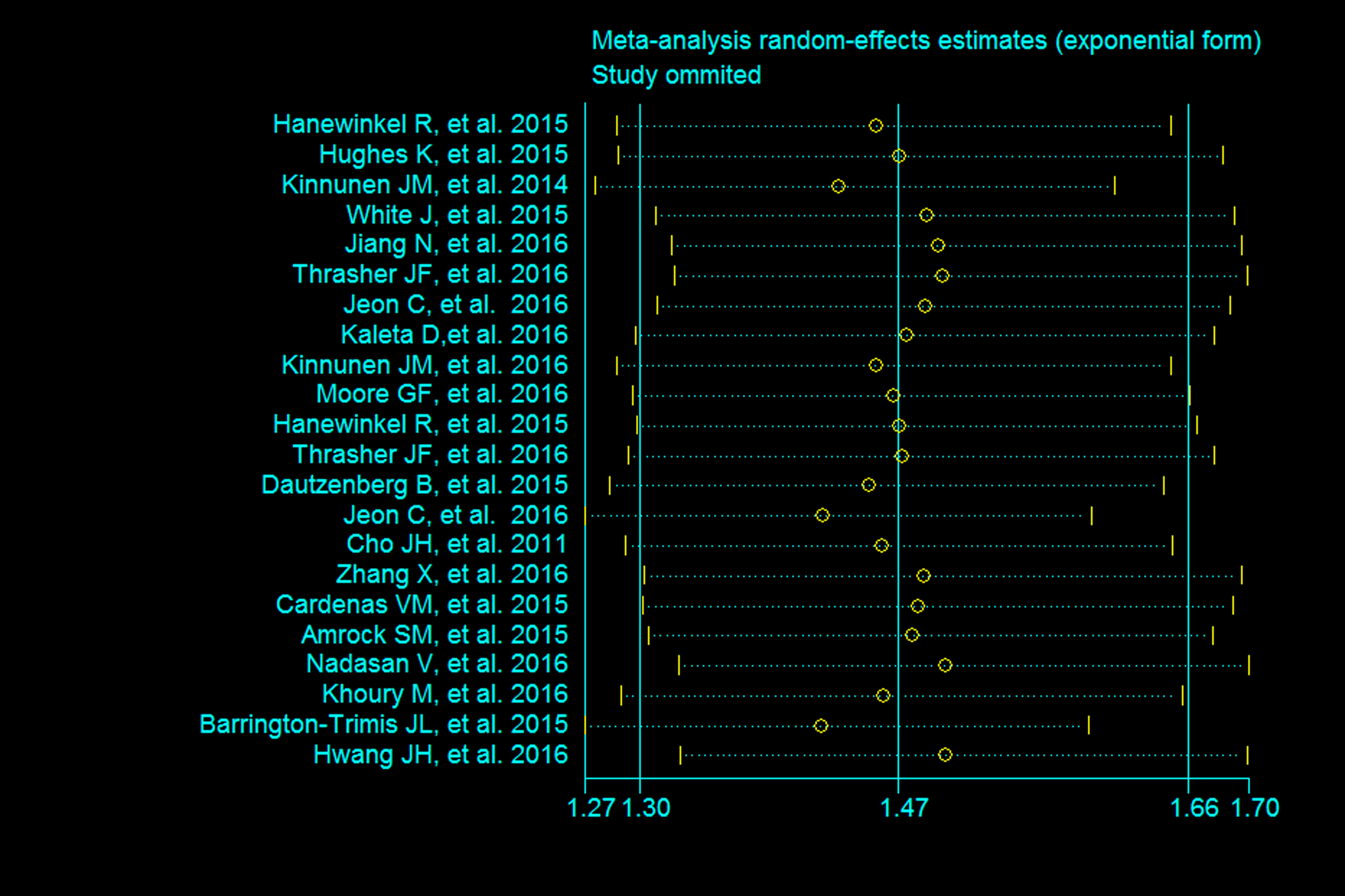

Supplement: Supplementary file 3 [file TID-16-05-s3.tif]

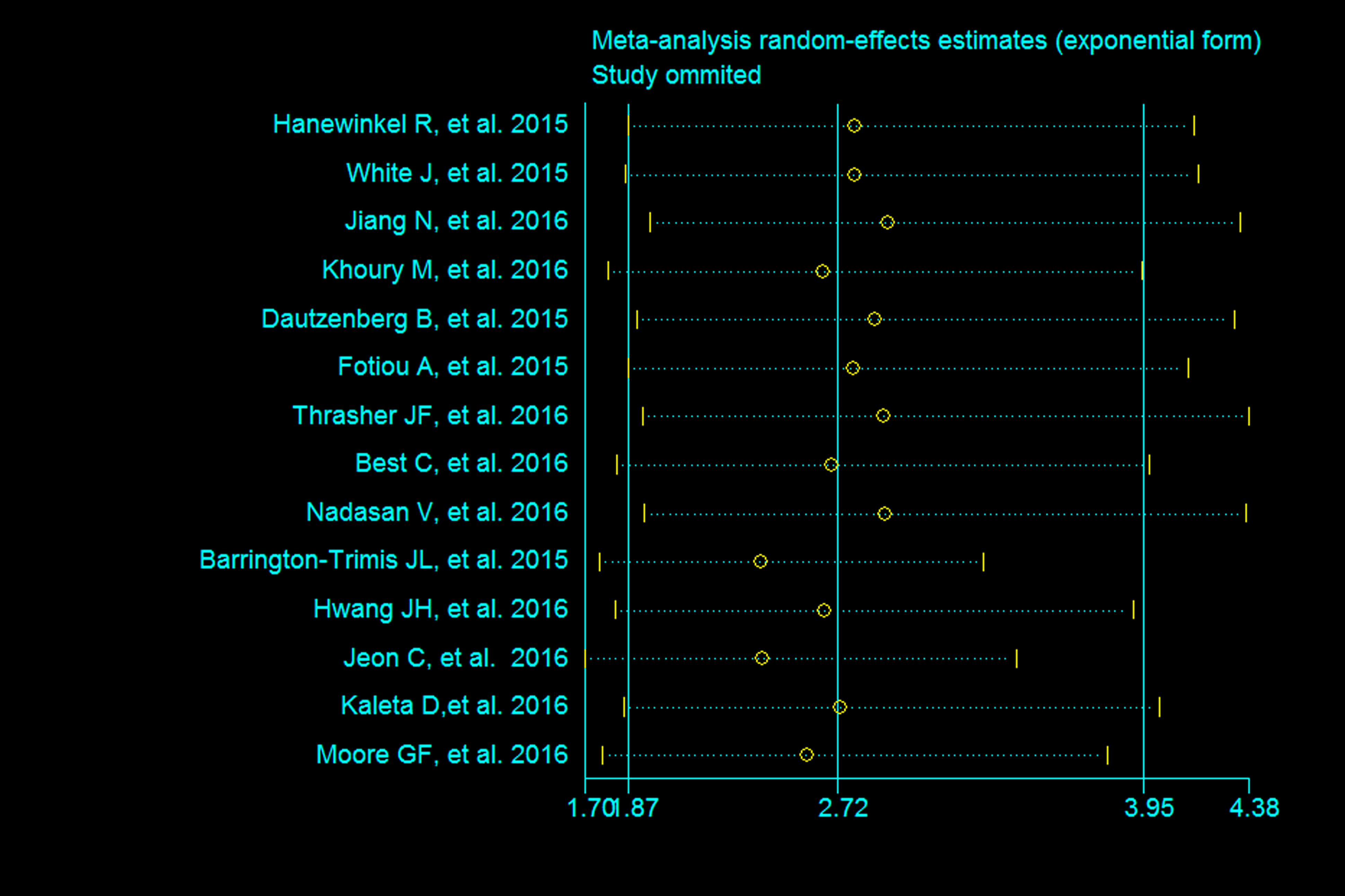

Supplement: Supplementary file 4 [file TID-16-05-s4.tif]
