# Supplementary material for: Smoking by family members and friends and electronic-cigarette use in adolescence: A systematic review and meta-analysis
Source: Tob Induc Dis. 2018 Feb 27;16:05. doi: 10.18332/tid/84864 (PMC6659504; doi:10.18332/tid/84864)
Supplement: Supplementary file 5 [file TID-16-05-s5.docx]

| *Table S1. Information of the studies included in the meta-analysis* | | | | | | |
| --- | --- | --- | --- | --- | --- | --- |
| Citation | Data source & age | Location | No. of e-cigarette users/participants | Study type | Electronic cigarette use definition | Variables adjusted |
| Hanewinkel et al.^25^ | Survey at 45 public secondary schools; Adolescents aged 11-15 years. | Germany | 126/2693 | Cross-sectional from a cohort study | Lifetime use of e-cigarettes was assessed by asking ‘Have you ever used an electronic cigarette?’ (yes or no). | Gender, age, sensation seeking, migration background, family affluence, at a gymnasium, experimental condition |
| Hughes et al.^26^ | 5th iteration of the Trading Standards North West  Alcohol and Tobacco Survey; School students aged 14-17 years. | North West  England | 3110/16193 | Cross-sectional | E-cigarette access was identified through a ‘yes’ response to the question ‘Have you ever bought or tried electronic cigarettes?’. | Gender, age, deprivation, smoking status, drinking status |
| Kinnunen et al.^13^ | 2013 Adolescent Health and  Lifestyle Survey; Adolescents aged 12-18 years. | Finland | 616/3535 | Cross-sectional | ‘Have you ever tried electronic cigarettes? How many times altogether?’ Options were: ‘No, I have tried once or twice’, ‘I have tried 20 times or less’, ‘I have tried more than 20 times’. | Age, gender, substance  (snus /waterpipe) use, smoking status, seen e-cigarette ads,  statement ‘smoking is for  loser’, school level, school  performance, family structure, father and mother’s work situation. |
| White et al.^27^ | Youth Insights Survey (YIS) 2014; Students aged  14-15 years. | New Zealand | 584/2919 | Cross-sectional | Respondents were asked ‘Have you ever tried electronic cigarettes? Those who answered ‘yes’ were classified as ‘e-cigarette ever-users’. | Gender, race, school decile  status, weekly income, smoking status, past month use of other tobacco products, marijuana , ever binge drinker. |
| Jiang et al.^28^ | School-based Survey on Smoking among Students 2012/2013 (75 secondary schools); Students with the average age of 14.8 years. | Hong Kong | 560/45857 | Cross-sectional | ‘In the past 30 days, which of the following products have you used: cigarettes, e-cigarettes, waterpipe, chewing tobacco, cigars, snus, smoking pipe or snuff, other tobacco product?’ Students who checked e-cigarettes were defined as current e-cigarette users. | Age, sex, knowledge about the harm of cigarette smoking and attitudes toward smoking, cigarette smoking, other tobacco use, alcohol consumption. |
| Khoury et al.^29^ | School-based study of grade 9 students; Students aged 14-15 years. | Ontario | 238/3312 | Cross-sectional | ‘Have you ever taken at least one puff from an electronic cigarette?’ (yes or no) | Sex, home smoking environment, smoking status of responders, family members, self-reported health and stress levels. |
| Dautzenberg et al.^30^ | School-based survey of schoolchildren aged 12-19 years. | Paris | 564/3279 | Cross-sectional | ‘Have you ever used an e-cigarette?’ (yes or no) | Age, gender, smoking status of father, mother, brother, sister; prohibition of tobacco use by one or two parents; ever use of tobacco, current smoking status, experimentation of shisha, cannabis; use of alcohol in the last month, experience of binge drinking; studying in a private or a public school, mean income per inhabitant of the school area. |
| Fotiou et al.^31^ | 2014 Health Behaviour in School-aged Children Survey (HBSC); Students aged 15 years. | Greece | 220/1320 | Cross-sectional | Lifetime use of electronic cigarettes was established from: ‘Have you ever used an electronic cigarette?’. Response options were: never, yes, only 1–2 times, yes more than twice but I don't use it now, yes more than twice and I use it now. | Gender, family context, tobacco and use of other substances. |
| Thrasher et al.^32^ | Survey at public middle schools; students aged 12-13 years. | Mexico | 1015/10146 | Cross-sectional | ‘Have you ever tried an e-cigarette?’ (yes or no) | Sex, age, students currently working, parents’ education, sensation seeking, alcohol use, drug use, technophilia, internet access in bedroom, internet tobacco and exposure, positive smoking expectancies, smoking status, perceived risks of e-cigarettes. |
| Best et al.^33^ | Survey at high schools; students with the average age of 14.0 years for Second 2 and 15.9 years for Second 4. | Scotland | 176/1020 | Cross-sectional | ‘Which one of the following is closest to describing your experience of e-cigarettes?’, to which they could respond ‘I have never used them’, ‘I have tried them once or twice’, ‘I use them sometimes (more than once a month)’ or ‘I use them often (more than once a week)’. This variable was dichotomised to ‘ever tried’ versus ‘never tried’. | Gender, age, family affluence scale, ethnicity. |
| Cho et al.^34^ | 2008 Health Promotion Fund Project; Students with the average age of 14.0 years for middle school and 16.5 years for high school. | Korea | 22/4341 | Cross-sectional | ‘Have you ever smoked an e-cigarette, even one or two puffs?’ If they answered in the affirmative, they were classified as having had the experience of e-cigarette use. | Gender, level of school,  propensity to be easily affected by friends, school life, cigarette smoking experience. |
| Zhang et al.^35^ | 2013 National Youth Tobacco Survey (NYTS); Students aged 11-18 years. | USA | 1466/18092 | Cross-sectional | In this study, we refer to e-cigarettes use as ever use of e-cigarettes. A student was classified as ‘ever used e-cigarettes’ if he/she reported ‘e-cigarettes’ to the question: ‘Which of the following tobacco products have you ever tried, even just one time?’ | Age, sex, race, cigarette smoking status, SHS exposure at home in past 7 days. |
| Cardenas et al.^36^ | 2012 National Youth Tobacco Survey (NYTS); Students aged 11-18 years. | USA | 451/22529 | Cross-sectional | Which of the following tobacco products have you ever tried, even just one time?  ‘Electronic Cigarettes or  E-cigarettes such as Ruyan or NJOY?’ were one of the eight types of  the choices under the instruction to choose all that applied. | Age, gender, race, smoking history |
| Amrock et al.^37^ | 2012 National Youth Tobacco Survey (NYTS); Students aged 11-18 years. | USA | 451/22529 | Cross-sectional | Students were asked, ‘Which  of the following tobacco products have you ever tried, even just one  time?’ to which they could select, ‘electronic cigarettes or e-cigarettes,  such as Ruyan or NJOY’ alongside other tobacco products. | E-cigarette harm perception, ever use of other tobacco products, tobacco products perception, sex, age, race |
| Nădăşan et al.^38^ | ASPIRE Romania study; Students with the average age of 14.9 years. | Romania | 707/1835 | Cross-sectional | E-cigarettes use was assessed by: ‘Which of the following products have you ever tried?’ Response options included ‘Yes’ or ‘No’ for e-cigarettes. | Sex, age, ethnicity, school type, school grade, perceived peer smoking, tried cigarette smoking, smoked cigarette in the past 30 days, sensation seeking, depressive symptoms, negative consequences, coping-reinforcement, social benefits |
| Barrington-Trimis et al.^39^ | 2014 Southern California Children’s Health Study; 11th- and 12th-grade students with the average age of 17.3 years. | California | 200/2,084 | Cohort | Participants who had used e-cigarettes on at least 1 of the past 30 days were classified as ‘current users’. | Gender, ethnicity, income, and highest parental education |
| Hwang et al.^40^ | 2013 Korea Youth Risk Behavior Web-based Survey; Adolescents aged 13-18 years. | Korea | 790/65753 | Cross-sectional | The e-cigarette use outcome variable was evaluated in two ways for current and ever e-cigarette use. First, ever e-cigarette use was defined as a ‘yes’ response to the following question: ‘Have you ever used e-cigarettes?’ Second, among ever smokers, participants who selected ‘yes’ to the question ‘During the past 30 days, have you used e-cigarettes?’ were considered current e-cigarette users. | Sex, school, location, perceived academic performance, frequency of alcohol drinking, experience of drug use, perceived stress level, cigarette smoking status |
| Jeon et al.^41^ | 2015 Yonsei Health Study; Middle and high school students aged 13-18 years. | Korea | 346/2744 | Cross-sectional | Current use of e-cigarettes was assessed by asking ‘During the last 30 days, have you used electronic cigarettes?’ (yes/no). | Sex, age, teachers smoking status |
| Kaleta et al.^42^ | Survey at second and high school students aged 13-19 years | Poland | 975/3552 | Cross-sectional | Those who have used e-cigarettes at least once during the past 30 days were classified as current e-cigarette users. | Gender, school grade, father/mother education, money available per month, alcohol use, tobacco smoking status, perception that tobacco smoking is harmful to health, e-cigarette harm perception, school has the rules restricting e-cigarette use, smoking ban in a school building and in the school area |
| Kinnunen et al.^43^ | 2015 Adolescent Health and  Lifestyle Survey; Adolescents aged12-18 years. | Finland | 101/6698 | Cross-sectional | Regular use was estimated only in 2015 with a question: ‘Which one of the following alternatives best describes your current use of e-cigarettes?’ with the options ‘I do not use e-cigarettes’, ‘I use e-cigarettes less than once a week, ‘I use e-cigarettes once a week or more often, but not daily’ and ‘I use e-cigarettes once a day or more often’. For the analysis of determinants for e-cigarette weekly use, answers to options ‘I use e-cigarettes once a week or more often, but not daily’ and ‘I use e-cigarettes once a day or more often’ were classified as weekly use of e-cigarettes. | Survey year, age, gender, snus/waterpipe use, smoking status, seen e-cigarette ads, academic achievement, family structure, father and mother’s work situation and parents’ education level. |
| Moore et al.^20^ | 2014 Child exposure to Environmental Tobacco Smoke (CHETS) Wales 2; Children aged 10-11 years. | Wales | 87/1495 | Cross-sectional | Electronic cigarettes use: Children were asked ‘Have you ever used an e-cigarette?’ with response options of ‘no’, ‘yes, once’ or ‘yes, more than once’. Children were classified as having used an e-cigarette if they responded ‘yes, once’ or ‘yes, more than once’. E-cigarettes were defined as electronic versions of cigarettes which do not give off smoke. | Sex, family affluence scale (FAS) |
